# Validation of a novel multi-exercise approach to isometric resistance training in normotensive adults

**DOI:** 10.1007/s00421-025-05785-3

**Published:** 2025-04-11

**Authors:** Ben H. Wright, Peter G. W. Jones, Mark R. Antrobus, Anthony W. Baross

**Affiliations:** 1 School of Sport, Nutrition and Allied Health Professions, Faculty of Health and Life Sciences, Oxford Brookes University, Oxford, UK; 2 Glasgow Caledonian University, Glasgow, Scotland; 3 School of Education, Childhood, Youth & Sport, Faculty of Wellbeing, Education and Language Studies, The Open University, Milton Keynes, UK; 4 Centre for Physical Activity and Life Sciences, Faculty of Art, Science and Technology, University of Northampton, Northamptonshire, UK

**Keywords:** Isometric exercise, Blood pressure, Handgrip, CR- 10 scale, Heart rate, Rate pressure product

## Abstract

**Purpose:**

Short- to long-term isometric resistance training (IRT) can produce clinically meaningful reductions in resting blood pressure, but established methods are costly or require laboratory access. An affordable method could improve accessibility; however, there is a need to establish efficacy and safety prior to prescription as an alternative IRT method. This study aims to determine whether a novel isometric training band (ITB) can elicit cardiovascular (CV) responses (blood pressure [BP] and heart rate [HR]) comparable with those of established methods.

**Methods:**

Fifteen normotensive adults (systolic [sBP]; 120 ± 3 mmHg, diastolic [dBP]; 71 ± 6 mmHg) completed a single 2-min isometric handgrip contraction (IHG) at 30% maximal voluntary contraction (MVC) followed by 2-min contractions for four individual ITB exercises at a self-determined intensity to replicate perceived exertion (CR-10) during IHG. A further 15 normotensive participants (sBP; 118 ± 6 mmHg, dBP; 68 ± 7 mmHg) completed bouts of IRT (IHG, 4 × 2 min at 30% MVC; ITB, 4 × 2 min at imposed CR-10 values [4–5]), with CV responses compared between bouts.

**Results:**

No differences in BP responses were detected between IHG and each ITB exercise (*P* > 0.05). CR-10 values and HRs were comparable between the individual IHG contraction and three ITB exercises (*P* > 0.05). Between bouts, regulating contraction intensity through imposed CR-10 values resulted in comparable BP responses (*P* > 0.05).

**Conclusion:**

These findings suggest that a novel ITB and associated protocol may serve as versatile, inclusive, and accessible alternative method for performing IRT.

## Introduction

Hypertension is a leading global preventable risk factor for all-cause mortality and cardiovascular-related disease (Zhou et al. 2021), with worldwide prevalence projected to affect 1.6 billion adults by the year 2025 (Kearney et al. 2005). As cardiovascular (CV) diseases are the leading cause of worldwide mortality (Cesare et al. 2024) with ischemic heart disease, cerebrovascular disease, and chronic kidney disease attributed to increased systolic blood pressure (sBP, ≥ 140 mmHg) (Bundy et al. 2017; Mills et al. 2020; Webb and Werring 2022), efficacious prevention, treatment, and management approaches are needed to address this global public health challenge. Modifications to lifestyle behaviors (diet, weight loss, reduced sodium consumption, and increased physical activity) form the initial management approaches of international hypertension guidelines (Unger et al. 2020; Whelton et al. 2018; Williams et al. 2018). Participation in physical activity and exercise are well-established interventions to reduce blood pressure (BP) (Islam et al. 2023; Naci et al. 2019; Noone et al. 2020) with aerobic exercise the preferentially recommended training mode (Ghadieh and Saab 2015). However, adherence to physical activity guidelines (150 min of moderate-intensity aerobic physical activity per week) remains low in adults (Guthold et al. 2016) and is further reduced for those with chronic conditions such as hypertension (Lopes et al. 2021). Thus, alternative, adherable, and accessible approaches are warranted.

Isometric resistance training (IRT) is an established non-pharmacologic approach in hypertension management (Edwards et al. 2024) given the reduced time requirements (~ 8 min per session, 24 min per week) and effectiveness of reducing resting and ambulatory BP irrespective of hypertensive status (Awuah and Dieberg 2023; Hansford et al. 2021; Smart et al. 2019) may serve as an complementary or alternative to traditionally prescribed aerobic exercise. Despite the potential benefits, there remains a reluctance for the wider prescription of IRT (Mancia et al. 2023). Acute bouts of exercise increase BP to meet tissue metabolic demands with this response exaggerated during isometric exercise (Greaney et al. 2015). Thus, safety concerns persist for broader application of IRT given a pronounced BP response to exercise is associated with cardiac events (Pescatello et al. 2015). However, comparable acute CV responses are observed between isometric exercise and dynamic resistance training (Kounoupis et al. 2020) when utilising low contraction intensities (30% maximal voluntary contraction). Studies have identified differing modes of IRT may elicit non-comparable BP responses, especially those of larger muscle mass (Coneglian et al. 2023a, b). Therefore, it is imperative that acute responses to novel IRT approaches are examined, and suitable methods are designed to regulate the CV response especially for methods utilising multiple and large muscle groups.

Effective IRT interventions have implemented various exercise modes, with isometric handgrip (IHG) completed thrice weekly (4 × 2-min contractions) at 30% of an individual’s maximum voluntary contraction (MVC), the most widely studied protocol (Smart et al. 2019). While alternatives such as bilateral leg extension exhibit BP-lowering capabilities (Baross et al. 2022; Inder et al. 2016; Wiles et al. 2010), this approach requires expensive, inaccessible, and non-portable equipment in addition to the requirement of repeat laboratory visits, compromising the time efficiency of IRT and imposing economic and accessibility participation barriers. Novel alternative IRT methods have been established, including the IsoBall (Baddeley-White et al. 2019) and isometric wall squat (IWS) (Lea et al. 2024; Wiles et al. 2017). However, with the equipment cost (~ £150) of the IsoBall, and the IWS requiring incremental exercise testing that necessitates the measurement of HR (Taylor et al. 2019), these methods still present accessibility barriers. To lessen the need for additional equipment and thus widening accessibility, studies have demonstrated the ability to self-regulate IRT using rating of perceived exertion (RPE) for both IWS (Lea et al. 2024) and IHG (Morrin et al. 2018). Furthermore, to mitigate cost, studies have evidenced BP reductions using inexpensive spring-loaded handgrip dynamometers (~ $2) (Millar et al. 2008). However, as modes (IWS and IHG) require the repetition of a single exercise, long-term adherence may be impacted by exercise monotony (Glaros and Janelle 2001) as autonomy of exercise choice and exercise versatility are positively associated with engagement (Teixeira et al. 2012; Wulf et al. 2014). Moreover, the IWS requires adopting a challenging exercise position, limiting accessibility for population groups presenting with musculoskeletal and mobility limitations. Thus, an inclusive option that enables exercise intensity to be regulated through RPE would further improve access to IRT. However, to date, no study has examined the viability of a cost-effective, inclusive, multi-exercise approach as an alternative method for IRT prescription.

Work undertaken from our laboratory has developed a novel isometric training band (ITB) designed to be inexpensive (~ £10) and user-friendly, with versatility to facilitate an array of isometric exercises capable of targeting larger and multiple muscle groups. The intended portability of the device was designed for future integration into home-based settings, allowing greater accessibility of IRT to the broader population with future incorporation into daily living (Collado-Mateo et al. 2021). However, as a novel approach, initial assessments are needed to establish viability, efficacy, and safety of the ITB to complete isometric exercise. Furthermore, to control for the exaggerated pressor response (Rowell 1993) likely to be elicited by the multi-exercise approach of the ITB, it is imperative to identify a suitable cost-effective method to regulate exercise intensity to ensure that the CV responses of the ITB remain within safe limits [> 250 mmHg sBP and/or > 115 mmHg dBP, or 85% age predicted maximal HR (Liguori 2020)].

Therefore, the aims of this study were to first determine RPE values during novel ITB exercises that replicate the CV demands of IHG exercise and second, to examine whether applying pre-determined RPE values during an ITB bout can elicit CV responses (BP, HR, and rate‒pressure product [RPP]) comparable with established IHG exercise without exceeding safety thresholds.

## Methods

### Research design

**Fig. 1:**
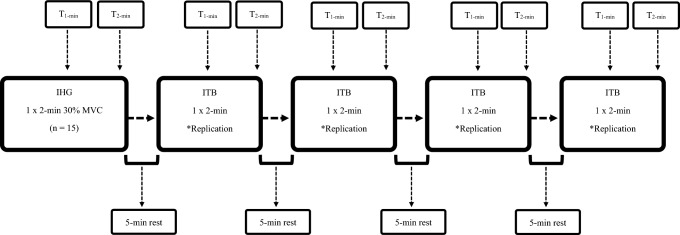
Schematic diagram of phase 1, including order of exercise, resting periods, and time points for measurement. T(_1-min_), 1 st minute, T(_2-min_), 2nd minute. IHG, isometric handgrip, ITB, isometric training band, MVC, maximal voluntary contraction. *Replication, self-determined perceived exertion comparable to IHG

**Fig. 2:**
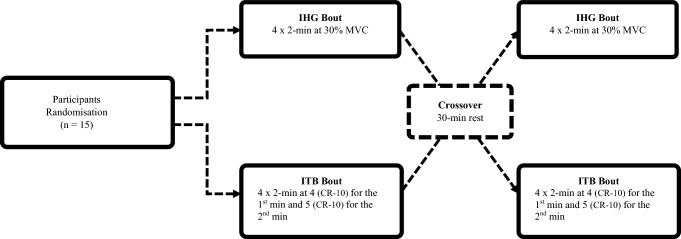
Schematic diagram of phase 2 including training group allocation, randomization, and training bout. IHG, isometric handgrip, ITB, isometric training band, CR- 10, Category-ratio scale

This study employed a sequential comparison design consisting of two phases. Phase 1 (Fig. 1) employed a quasi-randomized crossover within-subjects design to establish equivalent isometric training intensities for the ITB to the IHG using the Category Ratio scale (CR- 10; Borg 1998) and assessed the acute CV responses between exercises. Phase 2 (Fig. 2) utilised a randomized crossover within-subjects design to compare the CV responses between different bouts of IRT exercise to validate the ITB protocol to elicit CV responses comparable to IHG. To estimate sample size, a priori power analyses were completed via G*Power (v.3.1 Düsseldorf, Germany), with effect size measures (Cohen’s *d*) calculated (1.2) from previous acute response studies (Sagiv et al. 1988). The minimum sample size required was 8, with 15 recruited to account for anticipated dropout.

### Participants

Fifteen healthy normotensive (sBP; 120 ± 3 mmHg, diastolic [dBP]; 71 ± 6 mmHg) adults (12 males, 3 females; age, 26 ± 10.7 yrs; mass, 69.9 ± 13.1 kg; height, 172.1 ± 9.6 cm) were recruited; however, two participants were lost to follow-up between phases. Subsequently, an additional two participants were recruited for the second phase (sBP; 118 ± 6 mmHg, dBP; 68 ± 7 mmHg) adults (11 males, 4 females; age, 25.3 ± 9.6 yrs; mass, 74.4 ± 14.8 kg; height, 173.6 ± 9.5 cm). Participants were defined as normotensive from the baseline measurement of resting blood pressure (≤ 129 mmHg sBP and/or ≤ 84 mmHg dBp), with classifications based on the guidelines of the European Society of Hypertension (Mancia et al. 2023). Participants were excluded if classified as pre- or hypertensive (≥ 130 mmHg sBP and/or ≥ 85 mmHg dBP); if presenting with a diagnosis of cardiovascular, metabolic, or respiratory disease (type 1 or 2 diabetes; CVD; stroke; atrial fibrillation; chronic obstructive pulmonary disease); or if musculoskeletal impairment prevented the ability to complete isometric exercise. Prior to the initial visit, participants were provided with a copy of the participant information sheet outlining the study aims, procedures, potential risks, and benefits. Informed consent and a PAR-Q (PARQ+, 2020) were completed on the first visit to the laboratory. Institutional ethical approval was obtained (ETH1920 - 0152) from the University of Northampton Research Ethics Panel in accordance with the Declaration of Helsinki.

### Procedures

For each phase, participants visited the laboratory twice, with visits separated by a minimum of 24 h and a maximum of 72 h. The first visit included familiarization with the measurement protocols, training methods, and education on correct interpretation of the CR- 10 scale. Prior to each data collection visit, participants confirmed adherence to pre-testing requirements, which included avoidance of performing strenuous exercise within 24 h, abstaining from alcohol and caffeine for a 12-h period and to be fasted 4 h prior (Ahuja et al. 2009; Kallioinen et al. 2017).

### Blood pressure measurement

Resting and exercise BP (sBP, dBP, and mean arterial pressure [mBP]) were measured using a digital automatic BP device (Omron HEM- 907, Omron Healthcare, Japan). Resting BP was measured by placing the BP cuff covering 80% of the upper left arm approximately 1.5 cm above the antecubital fossa and with the artery position marker set in line with the brachial artery. Appropriate cuff sizing was identified following upper arm circumference measurement with the same cuff size used during subsequent measurements. During measurement, participants were seated with legs uncrossed, feet flat, and with their back supported, the upper arm was rested and supported at mid-sternal level. Clothing was non-restrictive, allowing access to the upper arm, and loose to prevent a tourniquet effect. Following 10 min of silent, seated rest, three BP readings were completed separated 1 min apart, with the mean value of the three readings used to determine baseline BP (Pickering et al. 2005). Exercise BP was measured immediately following the completion of each 2-min contraction (Richards et al. 2021) for each exercise mode, on completion of each contraction, participants were required to adopt the resting position and to remain still and silent during measurement.

### Measurement of heart rate

Resting and exercise HR (bpm^**·**− 1^) were recorded using a wireless HR chest strap (Polar FT1; Polar Electro Oy, Kempele Finland) and displayed continuously during exercise and rest, with values displayed at 1-s intervals. The chest strap was positioned in the center of the participants’ chest, with the two grooved electrodes moistened and the strap fastened firmly. Resting HR was calculated as the average of the final 30 s during 10 min of silent, uninterrupted, seated rest. Exercise HR was recorded as the average of the final 5 s during each min for each exercise.

### Rating of perceived exertion

Perceived exertion was assessed using the CR- 10 scale. Participants reported CR- 10 responses during the final 15 s of each min to IHG contraction and to each individual ITB exercise. The mean CR- 10 response for each min (T_1-min_ and T_2-min_) for each exercise were recorded. To compare CR- 10 responses between bouts, responses were averaged across each four IHG contraction, and each ITB exercise.

### Rate pressure product

Myocardial oxygen consumption (MV0_2_) was assessed non-invasively through calculating the rate pressure product (RPP, Gobel et al. 1978). Mean RPP values were measured for each isometric exercise by multiplying sBP by HR on completion of each 2-min contraction. Rate pressure product was calculated between exercise bouts by averaging the average RPP response to each IHG contraction and for each ITB exercise.

### Isometric handgrip

Isometric handgrip exercise was completed using a digital handgrip dynamometer (Zona Plus, Zona Health, USA). The device uses biofeedback to regulate 2-min alternating handgrip contractions at an intensity set at 30% of the participant’s MVC. Participants MVC was assessed through a 3 s maximal contraction following the American Society of Hand Therapists maximal grip strength protocol (Roberts et al. 2011). Once established, the device programmed 4 × 2-min handgrip contractions of alternating hands at 30% MVC starting with the right hand with a 1-min rest period provided between each individual contraction. Upon completion of a full training bout, a session was deemed successful if participants were able to complete at least 80% of the prescribed training session (Correia et al. 2020).

### Isometric training band

**Fig. 3:**
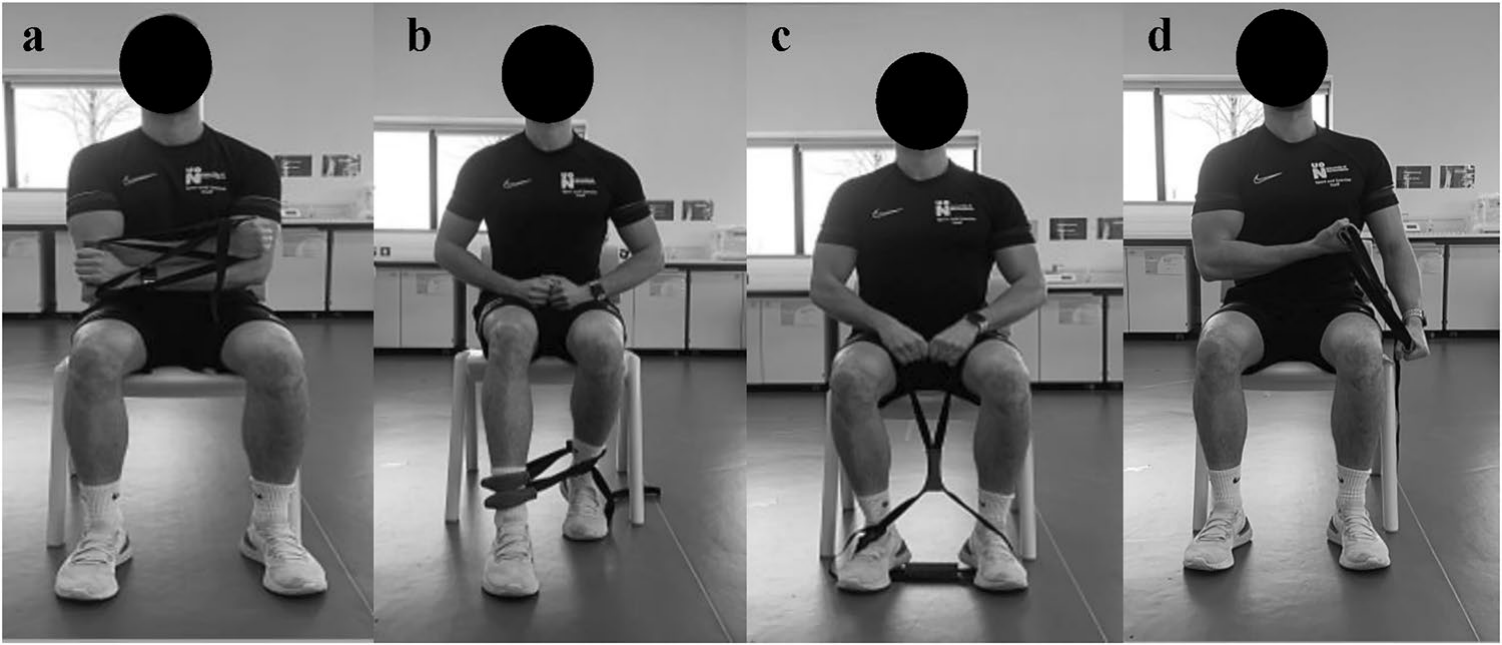
Participant positioning for isometric training band (ITB) exercises, **a** chest fly, **b** seated lunge, **c** seated pull, and **d** bicep curl/tricep extension

The isometric training band consisted of an adjustable polypropylene webbing figure of 8 with three rubber handle grips and a quick release buckle and ladder lock. Participants completed four exercises with the ITB consisting of a chest fly, seated lunge, seated pull, and bicep curl/triceps extension (Fig. 3). Each exercise was completed in a comfortable seated position with the back supported and feet placed shoulder width apart. Verbal instructions were provided throughout and to counter the possibility of imbalanced tension during each exercise, with participants instructed to apply equal amounts of force to each side of the band.

### Phase 1: Identifying equivalent CR- 10 responses between isometric exercises

To establish equivalent training intensities for the ITB to the IHG, participants reported CR- 10 responses during the final 15 s for each min of a single 2-min IHG contraction (Fig. 1). Participants then completed each of the four ITB exercises (chest fly, seated pull, seated lunge, bicep curl/triceps extension) in a randomized order at a self-determined intensity which best replicated the localized perceived exertion during the IHG contraction (Fig. 1). To support this, participants were instructed to report CR- 10 values following verbal instructions:

*“Consider how hard the exercise feels, consider the physical sensations occurring at contracting muscles. Focus on the effort, strain and fatigue, how much effort do you feel you are putting into the bands. Be as accurate as you can’’* (Modified from Morrin et al. 2018).

Between ITB exercises, participants remained seated and completed 5 min of silent, uninterrupted rest allowing CV values to return to baseline. Individual CR- 10 responses were recorded by averaging responses at the end of each minute (T_1-min_ and T_2-min_) for each isometric exercise in addition to CV responses (sBP, dBP, HR, and RPP) measured on completion of each 2-min exercise and averaged for each exercise.

### Phase 2: Comparing cardiovascular responses between exercise modes

To assess the CV response between IRT modes, participants were randomized to complete exercise bouts for the IHG and ITB (Fig. 2). For the IHG bout, participants completed 4 × 2-min contractions of alternating hands performed at 30% MVC, interspersed with 1-min rest periods. The IHG bout was deemed successful if the total session score was ≥ 80%. For the ITB bout, participants completed a 2-min contraction for each ITB exercise in a randomized order, interspersed by a 2-min rest period with contraction intensity regulated through imposed CR- 10 values. Participants were required to complete the first min (T_1-min_) of each exercise at an intensity corresponding to a CR- 10 value of 4, with the intensity increasing to a value of 5 upon completion of the second min (T_2-min_). An exercise anchoring procedure (Lagally and Costigan 2004) was applied to ensure participants understood ‘maximal’ on the CR- 10 scale, with participants required to complete a 3 s maximal contraction for each ITB exercise and explained that the sensation would correspond to a CR- 10 value of ‘10/maximal’, thus providing a reference point for the instructed pre-determined CR- 10 values. Between training bouts, participants silently rested undisturbed (30 min) until baseline BP and HR had returned to baseline values.

For each bout, HR and CR- 10 responses were recorded and averaged across each min of contraction (T_1-min_ and T_2-min_), with CV responses measured and averaged on completion of each 2-min contraction. The mean CV and CR- 10 responses were average across each ITB exercise and each IHG contraction, thus providing a mean of the means representing the overall response to each exercise bout. The CV response to each bout was deemed unsafe if BP values exceeded the ACSM, (2020) recommended exercise termination guidelines (Liguori 2020).

### Data analysis

Statistical analysis was completed using the Statistical Package of Social Sciences for Windows (SPSS, Version 28.0 IBM, Armonk, New York). Data were assessed for parametric assumptions (Field 2017) with normality of distribution analyzed through Shapiro‒Wilk tests. When the distribution of data was non-normal, log^10^ transformation was completed. For data that remained non-normal (dBP and HR), and for ordinal level data (CR- 10), non-parametric Friedman’s test was conducted to analyze differences across each exercise and bout with Bonferroni correction applied for post hoc assessment. Normally distributed data were analyzed through one-way analysis of variance with repeated measures (ANOVA) to examine differences in BP and CV responses between isometric contractions with Bonferroni tests used for post hoc analysis and partial eta-squared reported as the effect size (η_p_^2^). Between-bout differences and change data were assessed using paired samples t tests alongside Cohen’s *d* for effect size measures interpreted as small (0.2–0.49), medium (0.5–0.79), and large (> 0.8) (Cohen 1988), for non-parametric data the effect size measure *r* was calculated (Maher et al. 2013). Between-group variable data and change data were reported as mean ± standard deviation (SD). Results of pairwise comparisons were reported as mean ± sSD, mean absolute Δ ± SD, and *P* value, with α set at 0.05 for all analysis.

### Analysis of agreement

Limits of agreement (LoA) alongside Bland‒Altman plots were calculated between exercise bouts for sBP, dBP, RPP, and HR, with upper and lower 95% limits of agreement calculated with the formula

$${\text{Limits of Agreement}} = \left[ { \pm \left( {1.96 \times SD_{{{\text{of}}\;{\text{difference}}}} } \right)} \right]$$

with ($\overline{d }$) the mean difference and SD_of*difference*_ the standard deviation of observed differences (Ranganathan et al. 2017).

To examine agreement of the CV response between bouts, coefficient of variation (CoV) and intraclass correlation coefficients (ICC) were calculated for HR and each BP parameter for both the IHG and ITB, with ICC values being interpreted as ‘poor’ (< 0.50), ‘moderate’ (0.50–0.75), ‘good’ (0.75–0.90) and ‘excellent’ (> 0.90; (Koo and Li 2016). Pearson’s product-moment correlation coefficients (*r*) were calculated to examine the relationship between CV response between each IRT bout, and categorized as 0–0.1 negligible, 0.1–0.39 weak, 0.4–0.69 moderate, 0.7–0.89 strong, and 0.9–1.0 very strong (Schober and Schwarte 2018).

## Results

Adherence for each phase was 100% with participants successfully completing each isometric contraction and bout. For the IHG bout, participants achieved an average of 92.5% from the 80% requirement for maintaining IHG contraction intensity. In both exercise groups, no adverse events were reported.

### Perceived exertion between individual isometric contractions

**Fig. 4:**
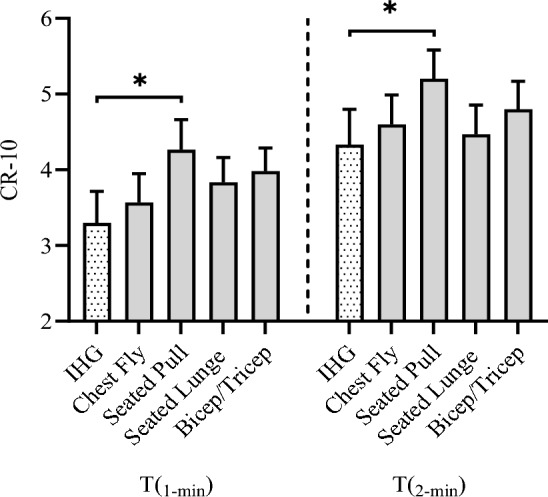
Minute CR- 10 responses to each isometric contraction (mean ± SE). T(_1-min_), 1st minute, T(_2-min_), 2nd minute *Significant difference to isometric handgrip (IHG) (*P* < 0.05)

Mean CR- 10 values for the single IHG contraction were 3.4 ± 0.4 for T_1-min_ and 4.4 ± 0.5 for T_2-min_, which were not significantly different for the chest fly (T_1-min_, 3.6 ± 0.3, Δ0.2 ± 0.7; Z = − 1.1, *P* = 0.157, *r* = − 0.2; T_2-min,_ 4.5 ± 0.4, Δ0.2 ± 0.8; Z = − 1.3, *P* = 0.206, *r* = − 0.3), seated lunge (T_1-min,_ 3.8 ± 0.3, Δ0.5 ± 1.1; Z = − 1.9, *P* = 0.054; *r* = − 0.5, T_2-min,_ 4.4 ± 0.3, Δ0.1 ± 1.1; Z = − 0.36, *P* = 0.726, *r* = − 0.1) and bicep curl/triceps extension (T_1-min_, 4 ± 0.3, Δ0.6 ± 1.1; Z = − 1.91, *P* = 0.063, *r* = − 0.5; T_2-min,_ 4.8 ± 0.3 Δ0.5 ± 1.4; Z = − 1.2, *P* = 0.236, *r* = − 0.3; Fig. 4). Reported CR- 10 scores were significantly different for the IHG and the ITB seated pull exercise χ^2^(9) = 61.002, *P* = < 0.001, with the seated pull values significantly greater than the IHG at both time points T_1-min_, (Δ0.8 ± 1.6; Z = − 2.2, *P* = 0.028, *r* = − 0.6), T_2-min_ (Δ0.9 ± 1.5; Z = − 2.3, *P* = 0.021,* r* = − 0.6; Fig. 4).

### Cardiovascular responses between individual isometric contractions

**Fig. 5:**
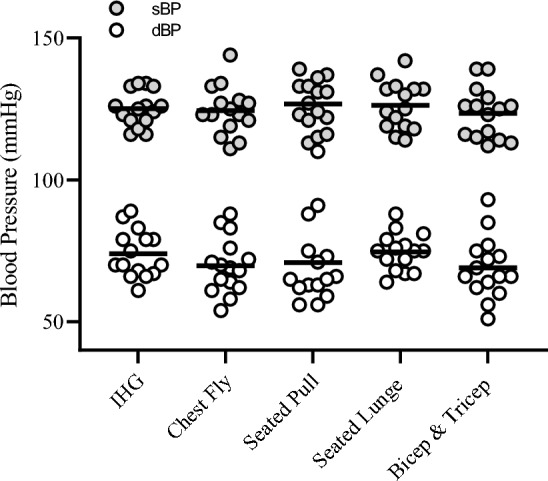
Mean and individual systolic (sBP) and diastolic (dBP) blood pressure responses to each 2-min isometric contraction for isometric handgrip (IHG) and isometric training band (ITB) exercises

Significant differences were observed between isometric contractions for RPP (*F*_(4,70)_ = 2.80, *P* = 0.032, η_p_^2^ = 0.138) HR at T_1-min_ (χ^2^(4) = 46.4 *P* = < 0.001) and at T_2-min_ (χ^2^(4) = 36.6 *P* = < 0.001) but not for sBP (*F*_(4,70)_ = 0.404, *P* = 0.805, η_p_^2^ = 0.023) or dBP (χ^2^(4) = 3.2 *P* = 0.161; Fig. 5). Pairwise comparisons identified RPP were significantly greater during the seated pull compared to IHG (10,153.4 ± 480.19 vs. 12,079.1 ± 481.46 bpm^**·**− 1^.mmHg, [Δ1925.7 ± 1506.2 bpm^**·**− 1^.mmHg]; *P* = 0.024). Similarly, the seated pull elicited significantly greater HR responses than IHG at T_1-min_ (76.5 ± 3.94 vs. 97.2 ± 4.56 bpm^**·**− 1^, [Δ20.7 ± 6.3 bpm^**·**− 1^]; *P* = 0.002) and T_2-min_ (81.1 ± 3.47 vs. 96.9 ± 4.49 bpm^**·**− 1^, [Δ15.7 ± 10 bpm^**·**− 1^]; *P* = 0.022).

### Cardiovascular responses between bouts

Participants successfully regulated ITB contraction intensity at the imposed CR- 10 values (T_1-min,_ 3.9 ± 0.2; T_2-min,_ 5.07 ± 0.48). Contraction intensity at this perceived exertion resulted in comparable cardiovascular responses between IHG and ITB, with no significant differences between bouts for sBP *t*_(14)_ = 1.28, *P* = 0.218, *d* = 0.32, dBP *t*_(14)_ = − 2.06 *P* = 0.058, *d* = − 0.53, and mBP *t*_(14)_ = − 1.30, *P* = 0.212, *d* = − 0.34, Table 1). Compared to IHG, the ITB bout elicited a significantly greater HR response *t*_(14)_ = 4.58, *P* = < 0.001, *d* = 1.15; 79.6 ± 2.57 vs. 90.7 ± 3.9 bpm^− 1^, [Δ 11.2 ± 9.4 bpm^**·**− 1^]), alongside a greater RPP *t*_(14)_ = 4.29, *P* = < 0.001, *d* = 1.08; 9995.1 ± 382.29 vs. 11,566.7 ± 521.02 bpm^**·**− 1^.mmHg, [Δ1571.6 ± 1417.7 bpm^**·**− 1^.mmHg]; Table 1).

**Table 1:** Cardiovascular responses between IHG and ITB exercise bouts

	IHG bout	ITB bout	Mean difference	CoV% (95% CI)	ICC (95% CI)
sBP (mmHg)	125 ± 3.4	127 ± 3	2.29 ± 6.6	2.8% [1, 4.6]	0.93 [0.80, 0.97]
dBP (mmHg)	72 ± 1.9	68 ± 2.5	− 3.4 ± 6.5	6.1% [4. 8.2]	0.8 [0.42, 0.93]
HR (bpm^1^)	79 ± 2.5	90 ± 3.9*	10.2 ± 8.8	9.2% [5.1, 13.4]	0.69 [− 0.18, 9.13]
RPP (bpm^**·**− 1^.mmHg)	9995 ± 382	11,566 ± 521*	1440 ± 1318	10.9% [− 666, 688]	0.90 [0.43, 0.93]

Data presented as mean ± standard error, IHG, isometric handgrip, ITB, isometric training band, CoV, coefficient of variation, (95%CI, lower and upper 95% confidence intervals). ICC, intraclass correlation coefficient. sBP, systolic blood pressure, dBP, diastolic blood pressure, HR, heart rate, RPP, rate pressure product

*Significant difference from IHG bout (*P* < 0.05)

### Agreement between bouts

**Fig. 6:**
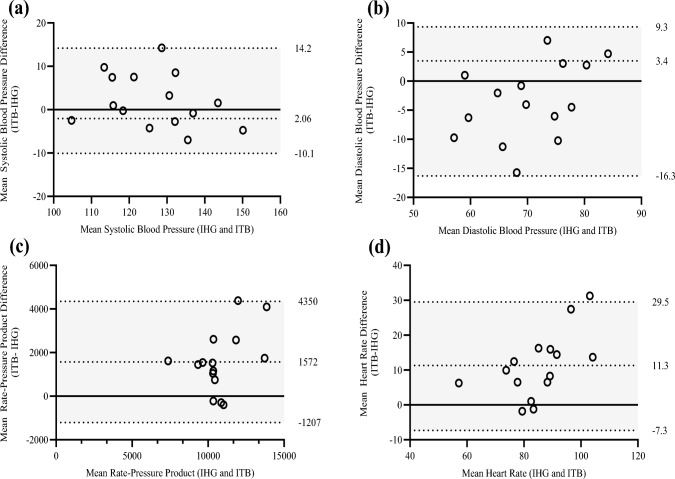
Bland–Altman plots for the IHG and ITB exercise bouts **a** systolic blood pressure, **b** diastolic blood pressure, **c** rate pressure product, **d** heart rate. The dashed central line represents the absolute average bias. Upper and lower limits are shown as dashed lines and represent ± 1.96 standard deviation

The between-bout CV responses were significantly positively correlated (sBP, *r* = 0.88; dBP, *r* = 0.75; HR, *r* = 0.80, RPP, *r* = 0.71, *P* < 0.001 across all measures), with acceptable LoA (sBP, $\overline{d }$ = 2.06 ± 6.1, 95% LoA [14.2, − 10.1 mmHg]; dBP, $\overline{d }$ = 3.48 ± 6.5, 95% LoA [9.3, − 16.3 mmHg]; HR, $\overline{d }$ = 11.13 ± 9.4, 95% LoA [29.5, − 7.31 bpm^**·**− 1^]; RPP, $\overline{d }$ = 1571 ± 633, 95% LoA [1207, − 4350 bpm^**·**−− 1.^mmHg] (Fig. 6**)**. The CoV for BP and HR ranged from 2.8% to 10.9% (sBP, 2.8%; 95% CI = 1, 4.6; dBP, 6.1%; 95% CI = 4, 8.2; HR, 9.2%; 95% = 5.1, 13.4; RPP, 10.9%; 95% CI = − 666, 688; Table 1).

Between-bout ICC values ranged from excellent (sBP: 0.93; 95% CI = 0.80, 0.97; *P* = < 0.001) to good (dBP: 0.80; 95% CI = 0.42, 0.93; *P* = < 0.001; RPP: 0.90; 95% CI = 0.43, 0.93; *P* = 0.002) and moderate (HR: 0.69; 95% CI = 0.18, 9.13; *P* = < 0.001; Table 1).

## Discussion

The aims of the present study were threefold: first to determine CR- 10 values for the ITB exercises which elicits comparable CV responses to IHG, second to establish if applying pre-determined CR- 10 values during an ITB bout produces equivalent acute CV responses to IHG, and finally to examine the magnitude of response to known safety thresholds. The findings suggest participants were able to complete each ITB exercise at a replicated perceived exertion that resulted in comparable CV responses to established IHG. Furthermore, this study revealed that with the exception of HR and RPP performing the ITB exercises with imposed CR- 10 values resulted in equivalent CV responses. However, at no point did any CV response exceed safety thresholds.

### Establishing equivalent CR- 10 responses between isometric exercises

The ability to self-regulate IRT contraction intensity is vital to ensure the stimulus is safe and effective, a consideration magnified for the ITB as the pressor response to exercise is augmented in proportion to the size of the exercising muscle mass (Coneglian et al. 2023a, b; Lewis et al. 1985; Seals et al. 1983). To control contraction intensity, IRT methods have typically required expensive laboratory-based equipment that allow the regulation of constant force (isokinetic and handgrip dynamometers), or constant electromyography (Gill et al. 2015; Wiles et al. 2010). However, for inexpensive methods that are unable to regulate force output, such as the ITB, RPE is a viable cost-effective alternative with potential application for increasing IRT accessibility. Participants reported equivalent CR- 10 values for each ITB exercise to those of the IHG which resulted in comparable BP responses with the exception of the seated pull (Fig. 4). As the seated pull requires a whole-body isometric contraction and thus significantly greater muscle mass than IHG, the larger CR- 10 response is unsurprising as studies have demonstrated RPE to relate to muscle activity (An et al. 2015), which is evident for both lower- and upper-limb exercise (Lagally et al. 2004; Duncan et al. 2006; Lagally and Costigan 2004). Moreover, RPE values are greater during bilateral than unilateral exercise at matched workloads (MacInnis et al. 2017). The recruitment of larger exercising muscle mass would have subjected participants to a greater CV demand heightening perceived exertion (Chen et al. 2002) evident with the larger HR and RPP response to the seated pull compared to the IHG across both time periods. The significant increase in HR is consistent with studies examining the acute CV responses to isometric contraction (Ash et al. 2017; O'Driscoll et al. 2021). Therefore, it is plausible that larger exercising muscle mass leads to greater motor unit recruitment and firing frequency, increasing corollary signaling heightening perception of effort (Cafarelli 1982) in addition to somatosensory feedback from larger active muscle mass activating both mechano- and metaboreceptors, further contributing to the increased perception of effort (Pageaux 2016). Consequently, as BP responses were comparable between contractions, it was deemed appropriate to apply the corresponding CR- 10 values during a complete ITB exercise bout.

### Comparing cardiovascular responses between different isometric exercise modes

With an exaggerated pressor response elicited during isometric exercise (Rowell 1993) alongside exercise-induced increases in sBP associated with cardiac events (Mariampillai et al. 2020; Pescatello et al. 2015), it is paramount that the safety of novel IRT methods is established prior to wider application. The lack of difference in the BP responses between bouts suggests applying the pre-determined CR- 10 values to the ITB exercises can adequately provide a safe and comparable BP response equivalent to efficacious IRT methods (Edwards et al. 2022). On review of individual data, peak BP responses (calculated as the highest recorded value within each group) during the ITB bout (sBP, 147 mmHg, dBP, 86 mmHg) did not exceed exercise termination thresholds (> 250 mmHg sBP and/or > 115 mmHg dBP; Liguori 2020) and are similar to those reported during IHG (sBP, 150 mmHg, dBP, 92; Carlson et al. 2017), isometric single arm flexion (sBP, 158 mmHg, dBP 91 mmHg), and isometric single leg extension (sBP, 158 mmHg, dBP, 94 mmHg; Wiles et al. 2005). These findings suggest the application of imposed CR- 10 values for the novel ITB approach allows contraction intensity, and therefore the CV response, to be safely controlled to the extent that the stimulus is equivalent to established IRT methods.

The ITB bout did elicit a larger HR and RPP response compared to that of the IHG though a likely consequence of the size of the exercising muscle mass (Gálvez et al. 2000) given both upper- and lower-limb bilateral exercises are incorporated within the ITB protocol. Studies indicate that the size of the exercising muscle mass influences the magnitude of the CV response to acute isometric exercise (Coneglian et al. 2023a, b). The heightened pressor response during isometric contraction is mediated by an augmented cardiac output through a rise in HR (Rowell 1993), which explains the significant increases observed during the ITB bout. It is expected the multi-exercise approach would result in greater systemic blood flow occlusion leading to a greater HR response to maintain adequate perfusion to the exercising muscle tissue (Murphy et al. 2011). Additionally, whole-body and bilateral exercises result in greater rate of metabolic accumulation (e.g., lactate, H^+^, and Pi) (Migiano et al. 2010) and mechanical distortion, leading to increased activation of group III/IV afferent fibers (Rossman et al. 2014), subsequently producing a reflexive cardiovascular response (Seals 1989). Importantly, the data indicate peak HR values during both exercise modes (IHG, 97 bpm^**·**− 1^; ITB, 118 bpm^**·**− 1^) were significantly lower if ACSM exercise termination guidelines (Liguori 2020) were applied to the present sample (~ 165 bpm^**·**− 1^) though the criterion for achievement of 85% age predicted maximal HR is disputed (Jain et al. 2011) and considered a poor predictor for adverse cardiac events (Whitman and Jenkins 2021).

In contrast, RPP reflects MV0_2_ and is a recognized indicator of myocardial load (Jiang et al. 2023) which is strongly associated with future adverse clinical events (Jiang et al. 2023; Whitman et al. 2019), thus a more meaningful marker to determine the safety of exercise response. Although RPP was significantly greater during the ITB bout, peak values across each mode (IHG, 12,848 bpm^**·**− 1^.mmHg; ITB, 15,903 bpm^**·**− 1^.mmHg) were lower than maximal thresholds during exercise stress testing (≥ 25,000; Fletcher et al. 2001). RPP during the ITB bout was lower than values are evidenced within cardiac rehabilitation (17,304 bpm^**·**− 1^.mmHg; Adams et al. 2010) and during treadmill or bicycle ergometry in hypertensives cohorts (Abiodun et al. 2015). Also, values were lower than those of IRT utilising larger muscle mass in hypertensives (20,681 bpm^**·**− 1^.mmHg; Wiles et al. 2018) to which authors reported no adverse events. As the ITB is a uniquely devised training approach, this is the first study to examine its feasibility and safety for completing isometric exercise. Collectively, these findings suggest the ITB and the associated protocol can adequately elicit a safe and comparable CV response to established methods, and thus support its application as a novel IRT approach.

A limitation of the present study is that findings are limited to a healthy, normotensive population, and given that hypertensive status influences the magnitude of the CV response to isometric contraction (Carlson et al. 2017), and findings are not applicable to pre- and hypertensive adults. Consequently, future research is required to establish safe and effective methods of intensity regulation for novel IRT across pre- and hypertensive cohorts. Additionally, studies with larger sample sizes are required to assess the ability to reliably reproduce CV responses during novel ITB exercises utilising imposed RPE values.

## Conclusion

For the development of a novel, inexpensive, and accessible IRT approach, this study forms the essential foundation in establishing the feasibility of the ITB and associated protocol. Findings suggest utilising imposed CR- 10 values during ITB exercises can sufficiently elicit a safe, CV demand that is commensurate with established methods. However, the use of the ITB to reduce BP is yet to be examined; therefore, further research is required to assess the efficacy of the ITB and the associated protocol as an anti-hypertensive training approach.

## Data Availability

The datasets generated during and/or analysed during the current study are available from the corresponding author on reasonable request.

## Abbreviations

ANOVA: Analysis of variance
BP: Blood pressure
BPM: Beats per minute
CR- 10: Category-ratio scale
COV: Coefficient of variation
CV: Cardiovascular
DBP: Diastolic blood pressure
LOA: Limits of agreement
MVC: Maximum voluntary contraction
MBP: Mean arterial pressure
MIN: Minute
MMHG: Millimeters of mercury
MV0_2_: Myocardial oxygen consumption
ICC: Intraclass correlation coefficient
IHG: Isometric handgrip
IRT: Isometric resistance training
ITB: Isometric training band
RPP: Rate pressure product
S: Seconds
SBP: Systolic blood pressure
